# Neuralgia of the Urethra

**Published:** 1844-03-23

**Authors:** 


### NEURALGIA OF THE URETHRA.

A woman, thirty-two years of age, mother of four children, suffered for eight months from pain at the lower part of the abdomen, with scalding on making water, and a constant sense of titillation at the orifice of the meatus. The pain became so severe as to prevent the patient from sleeping. The bladder was examined, but no sign of calculus found. Various remedies were tried without effect. Two issues, with the Vienna caustic, were now made over the hypogastric region. The patient had tepid baths, containing two drachms of the sulphate of potass, and some pills composed of hyosciamus and extract of lettuce. This mode of treatment effected a cure. —*Bordeaux Journal.*

